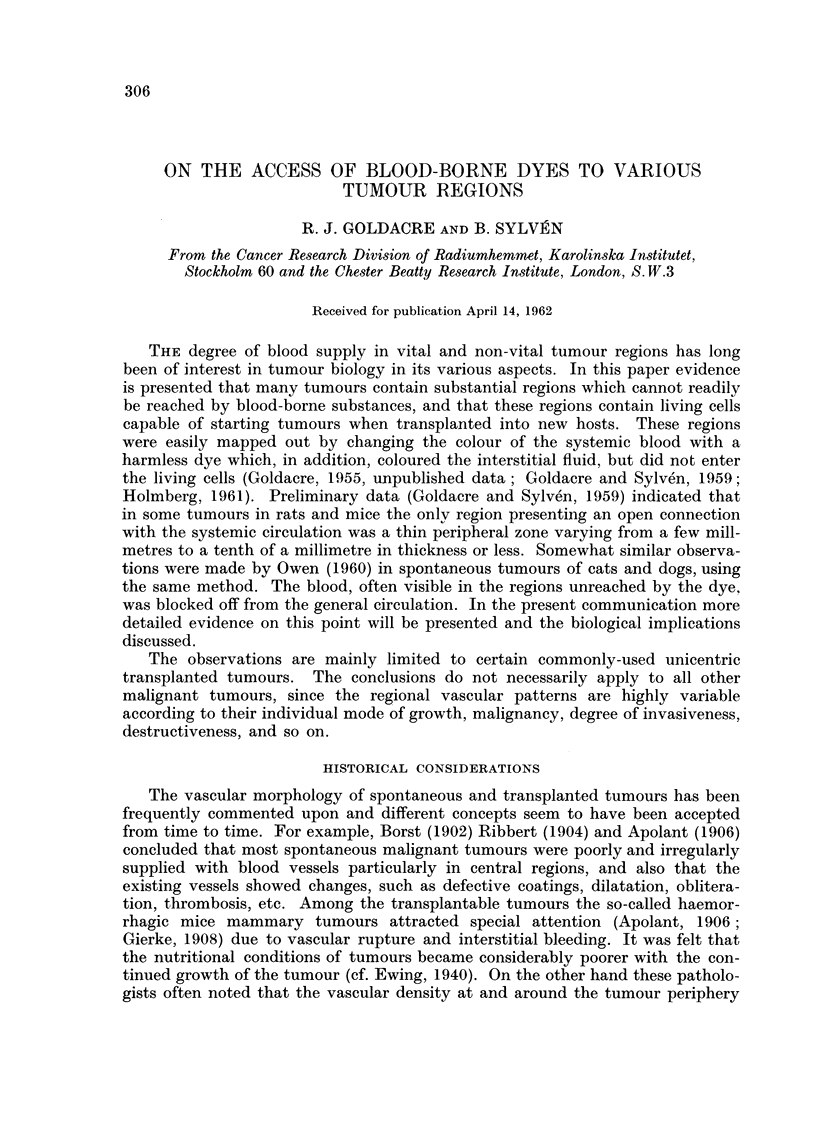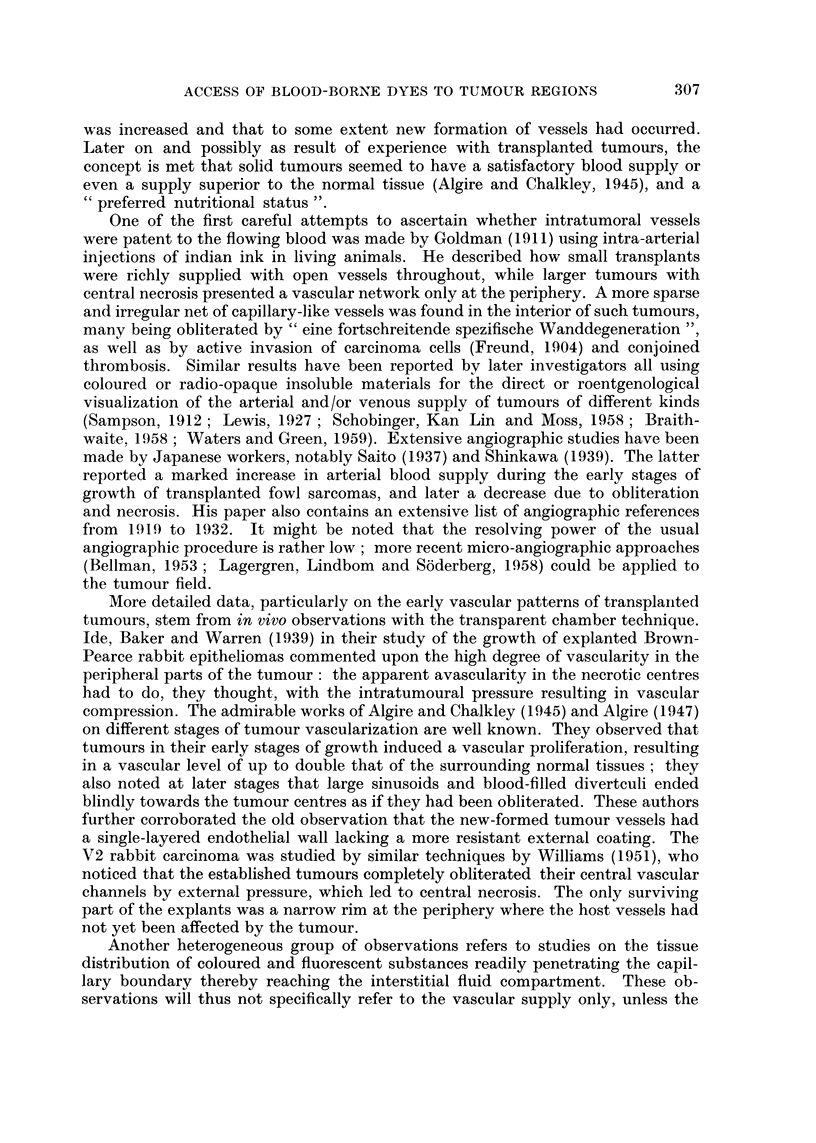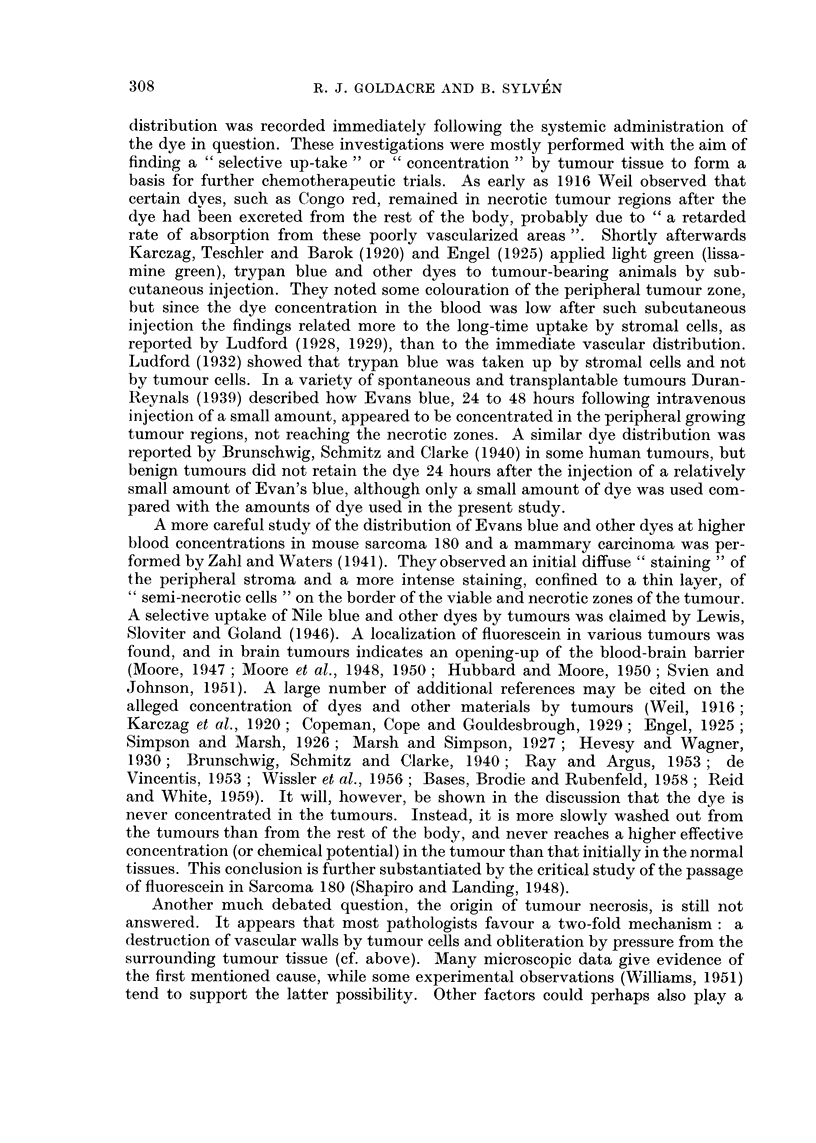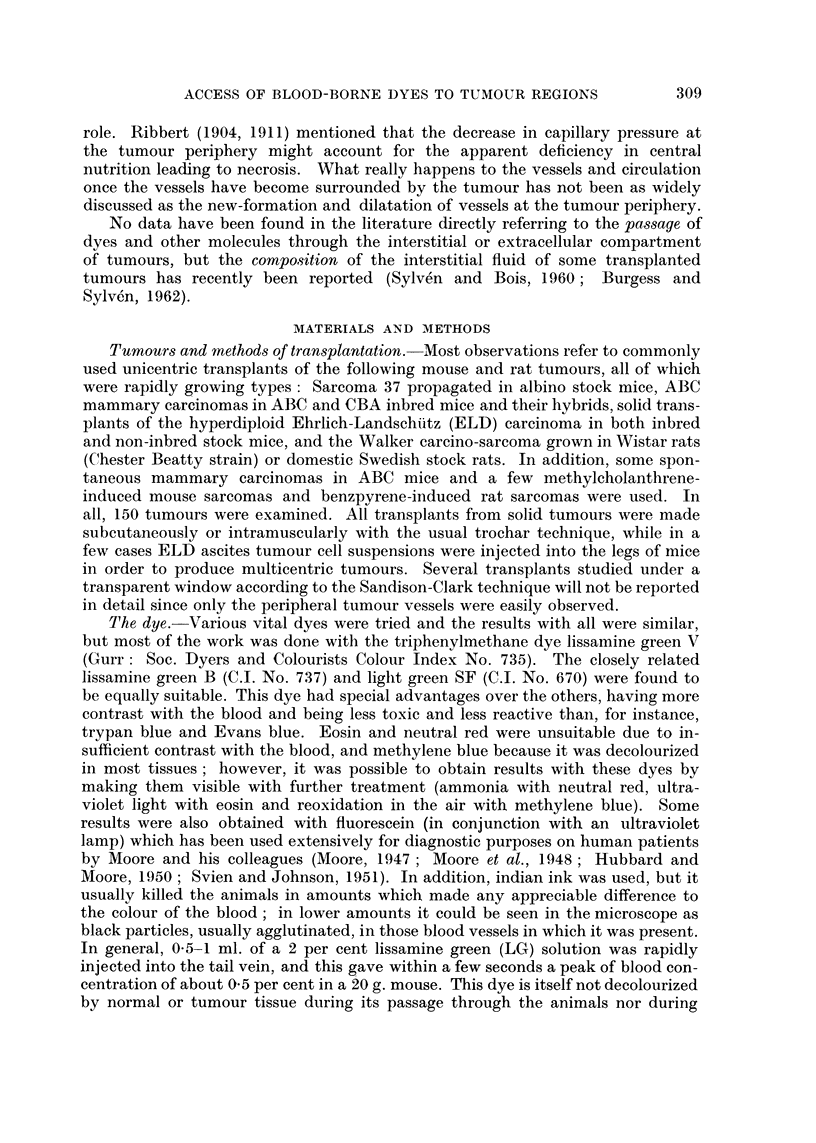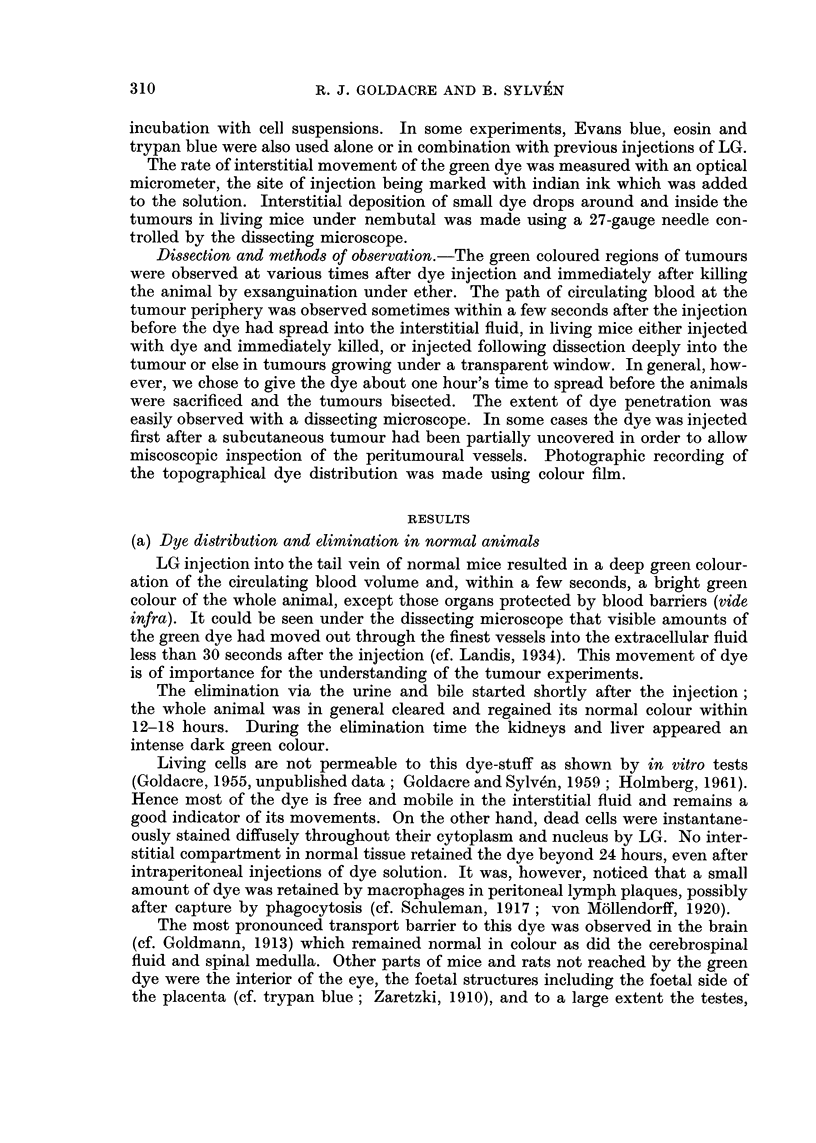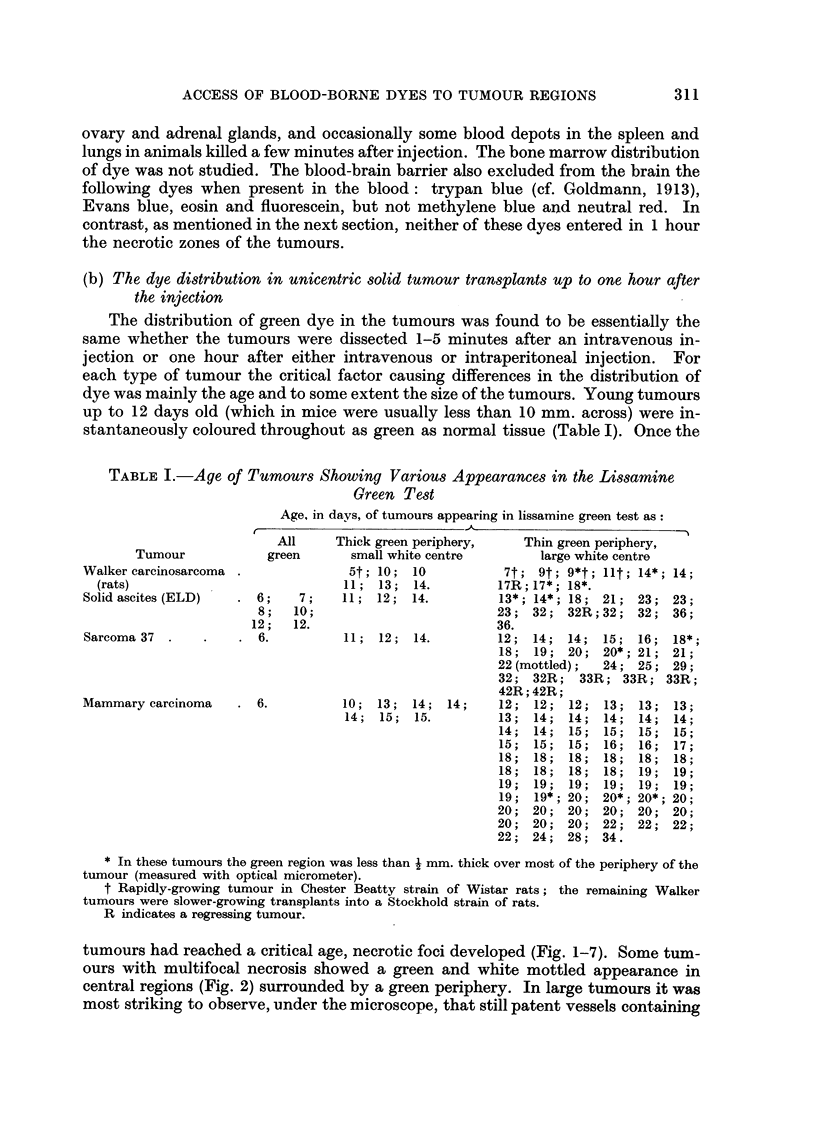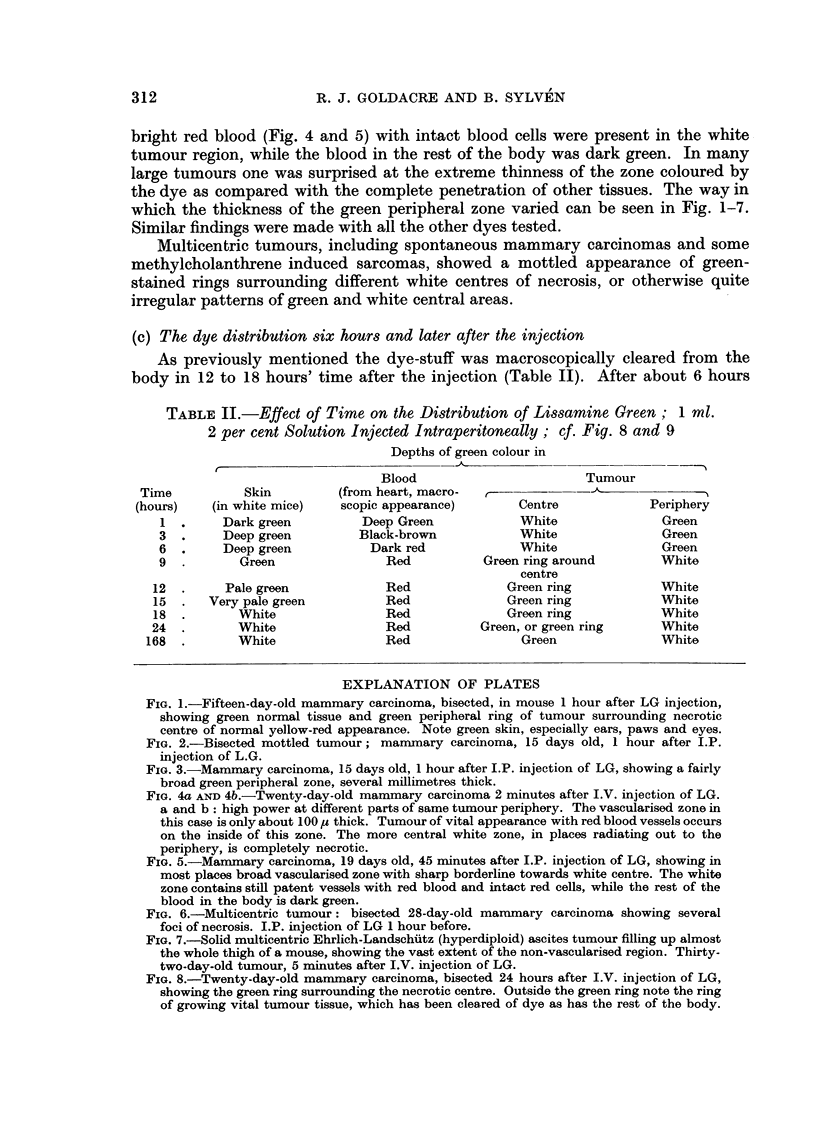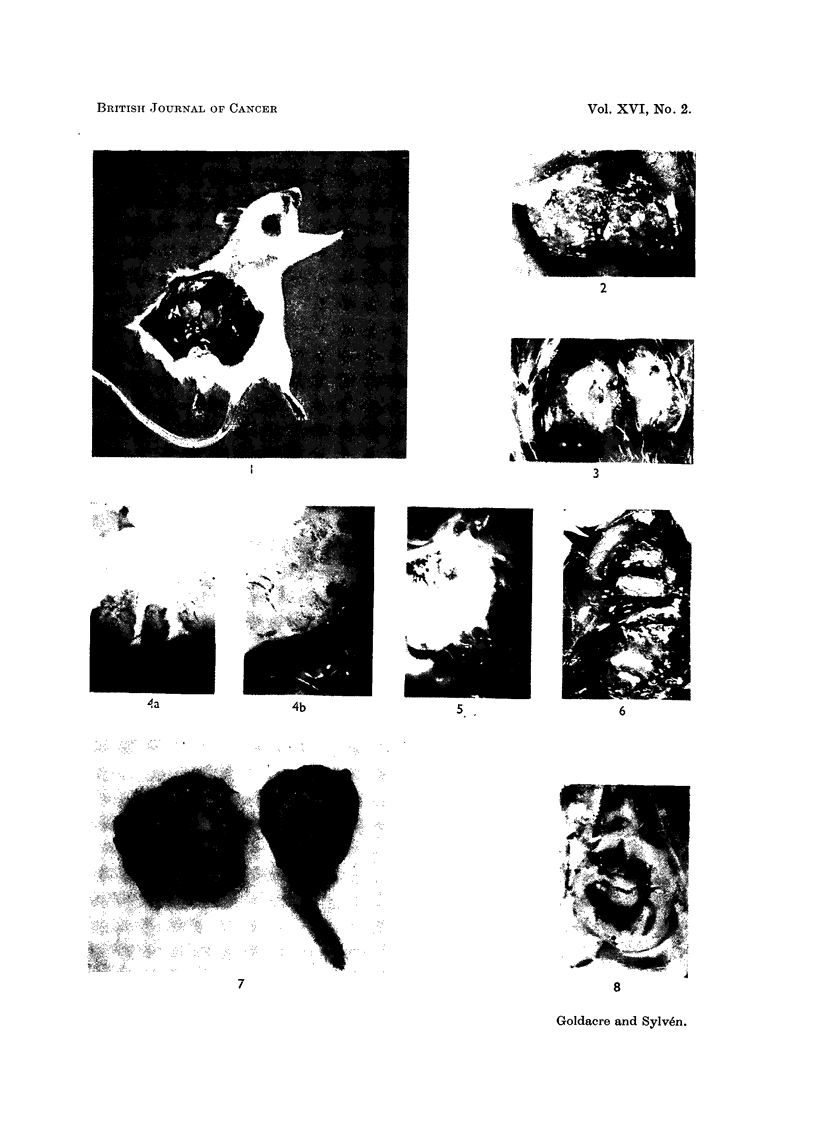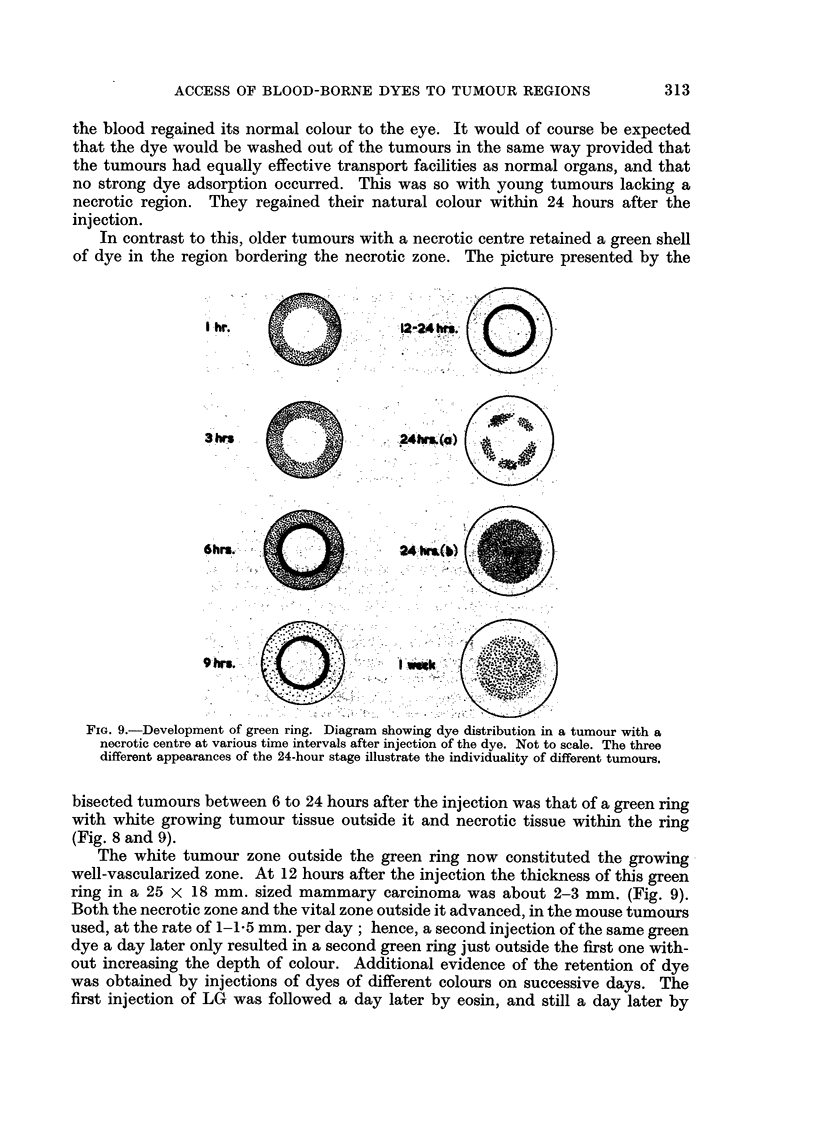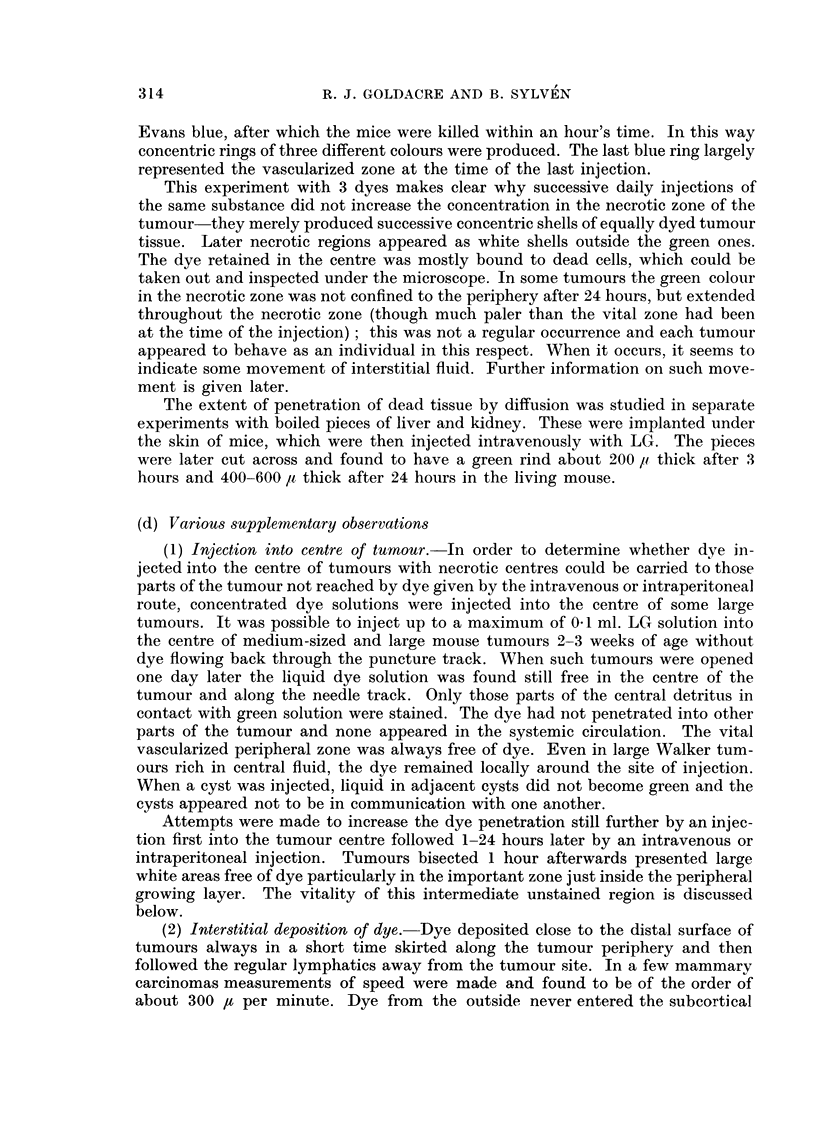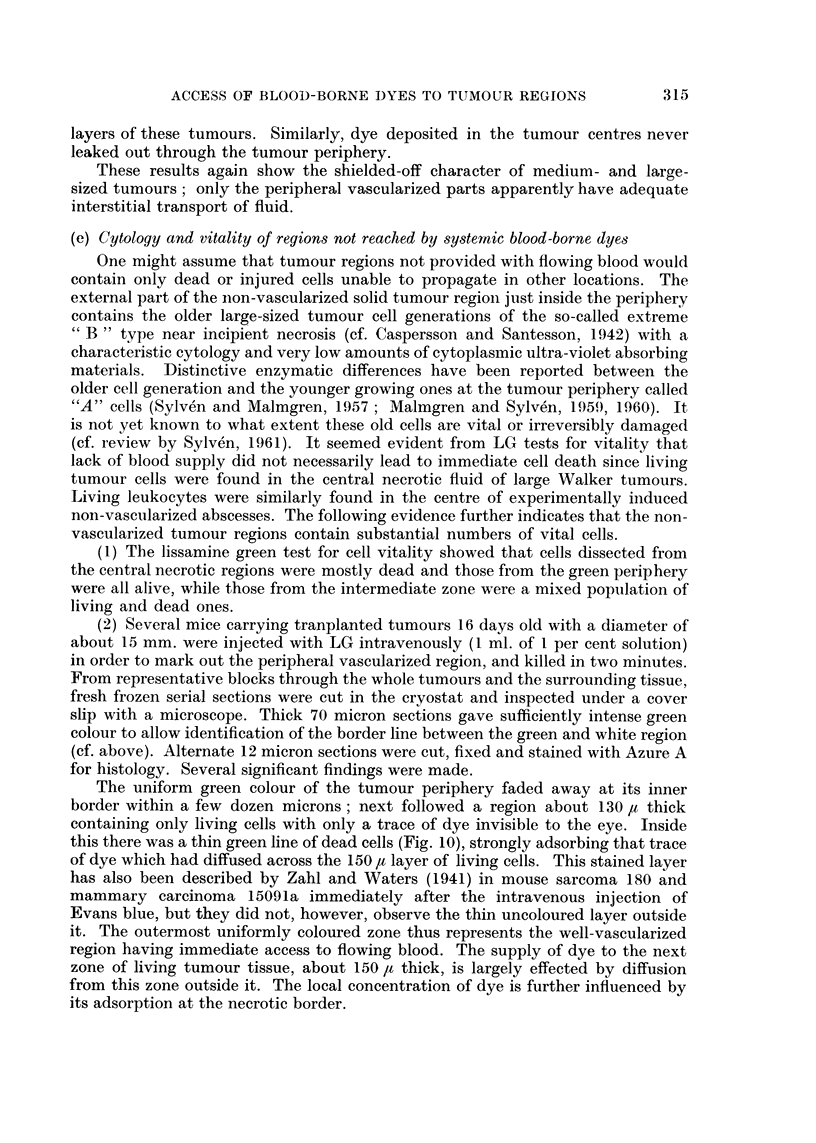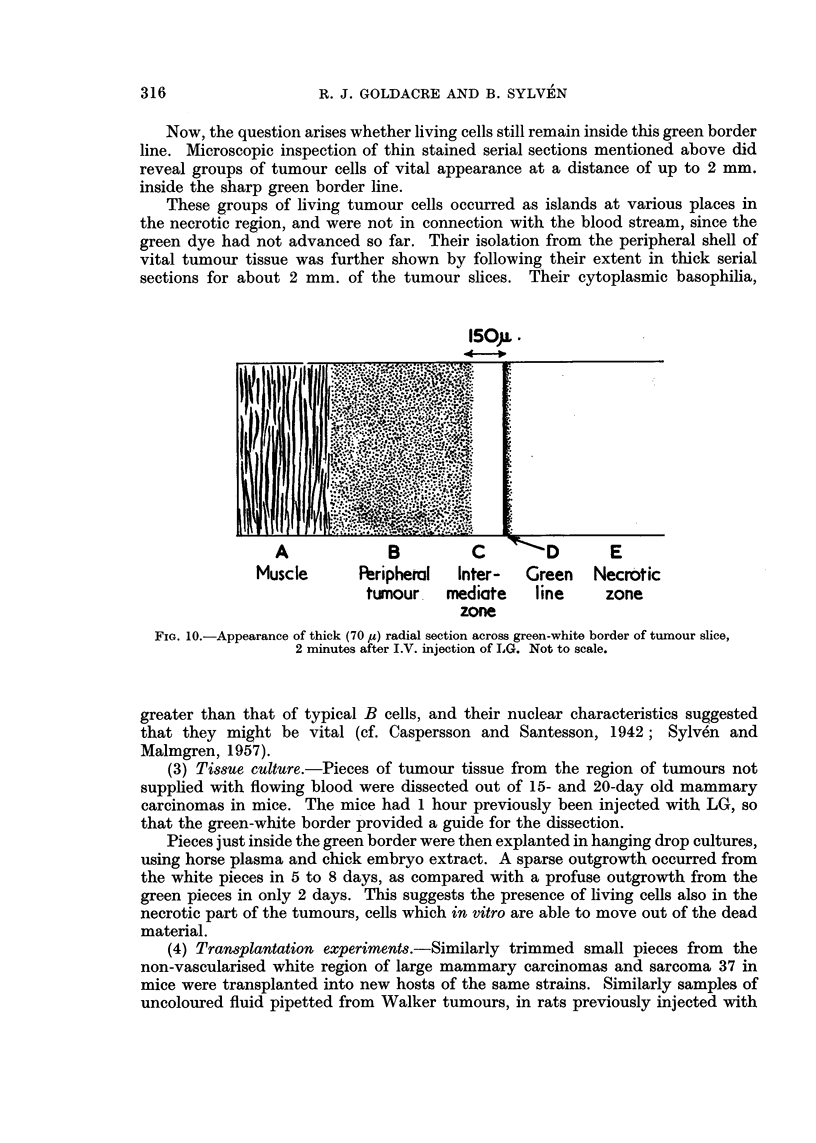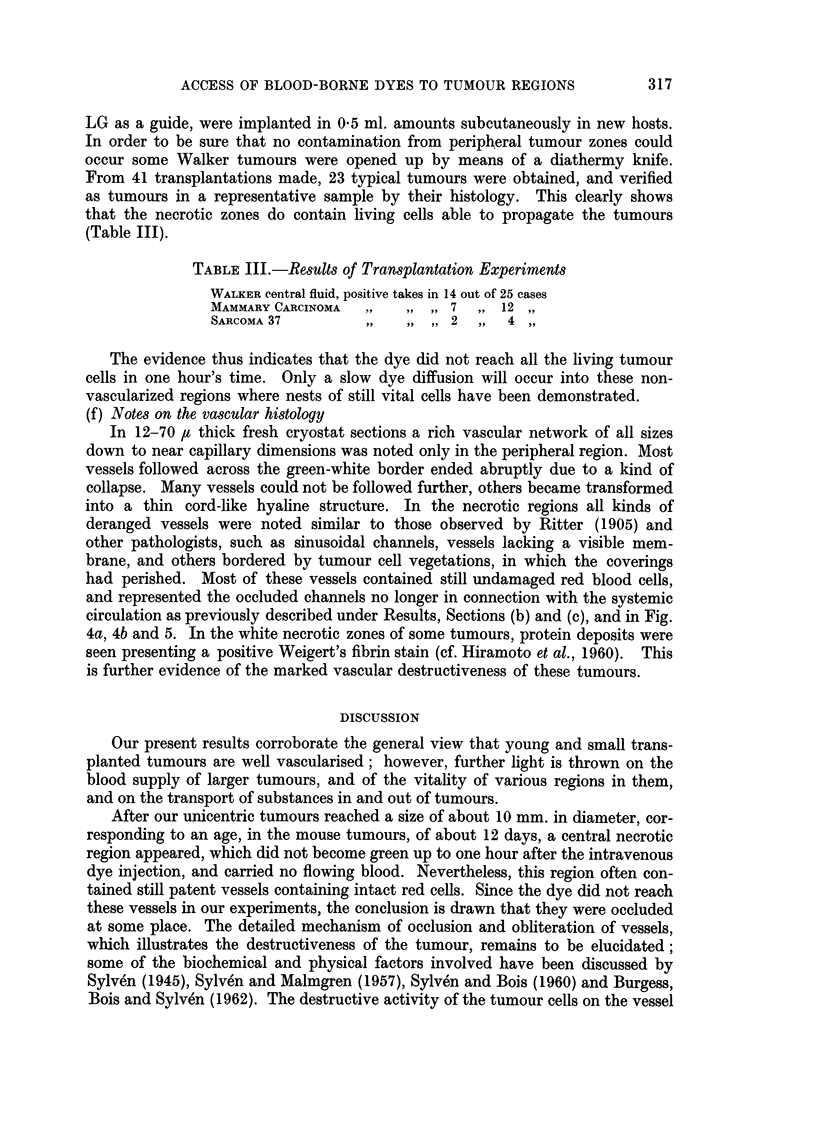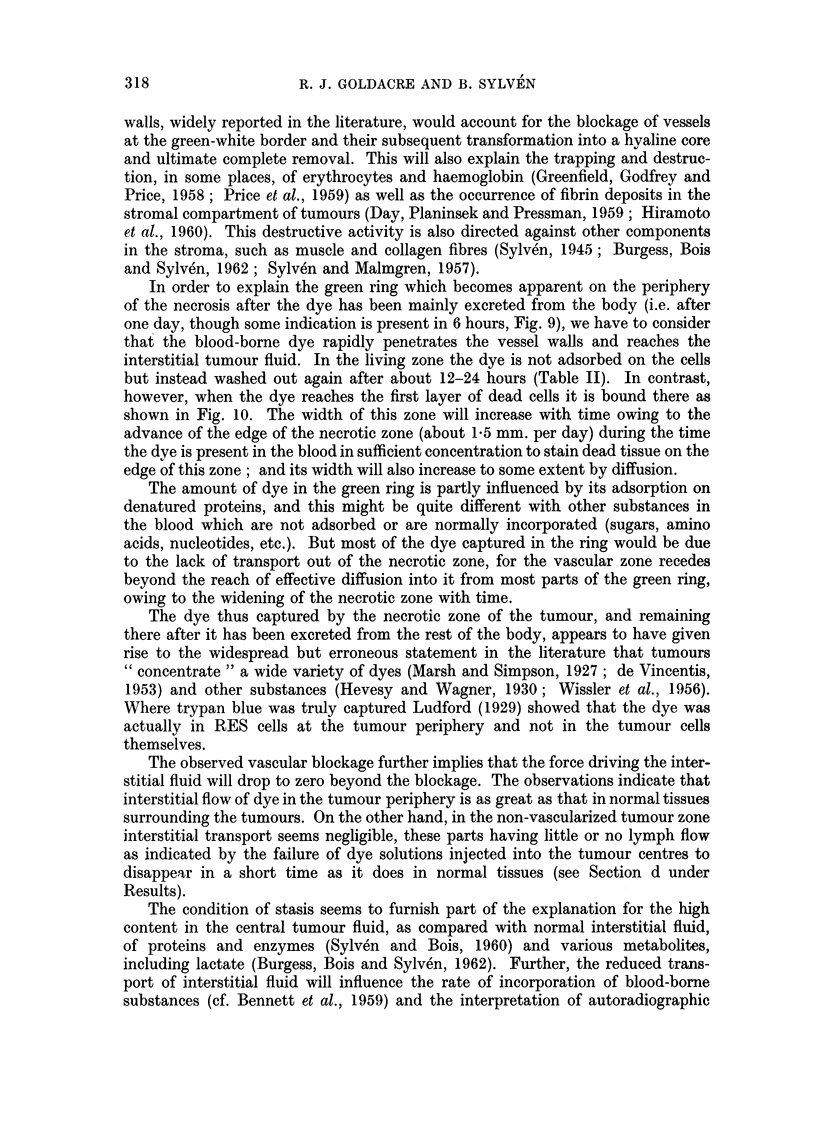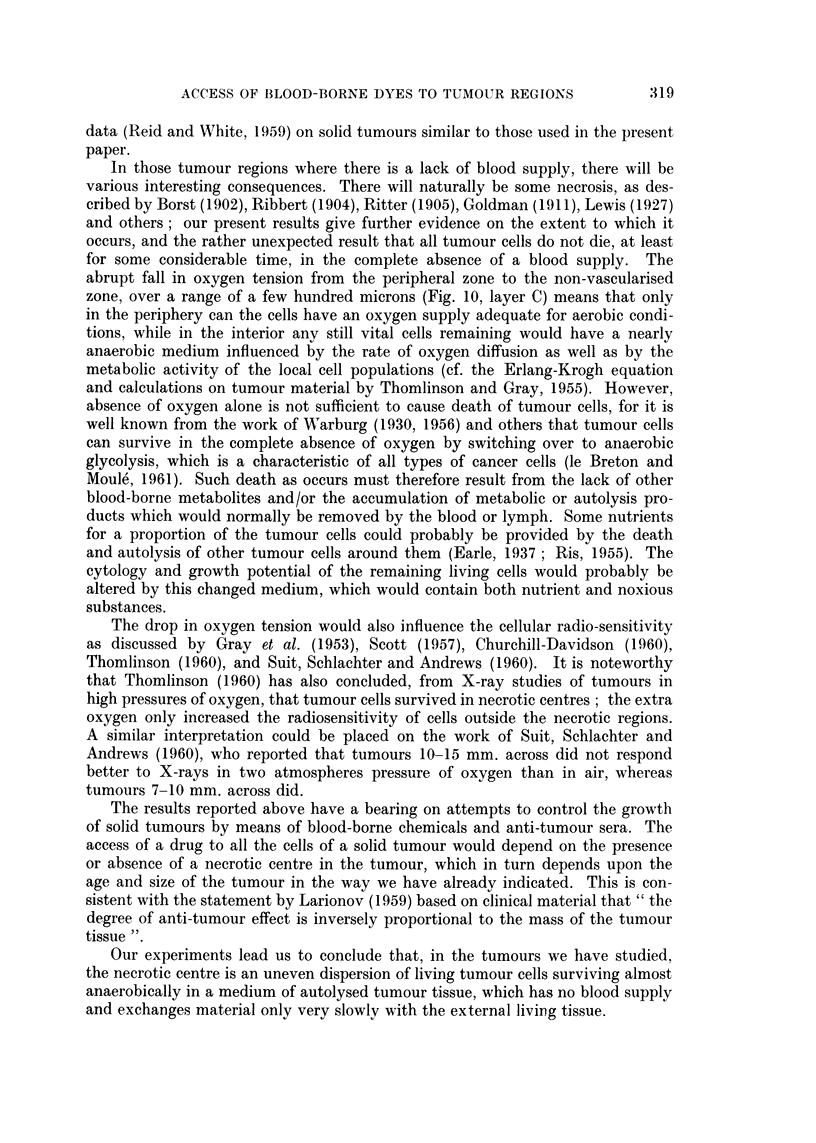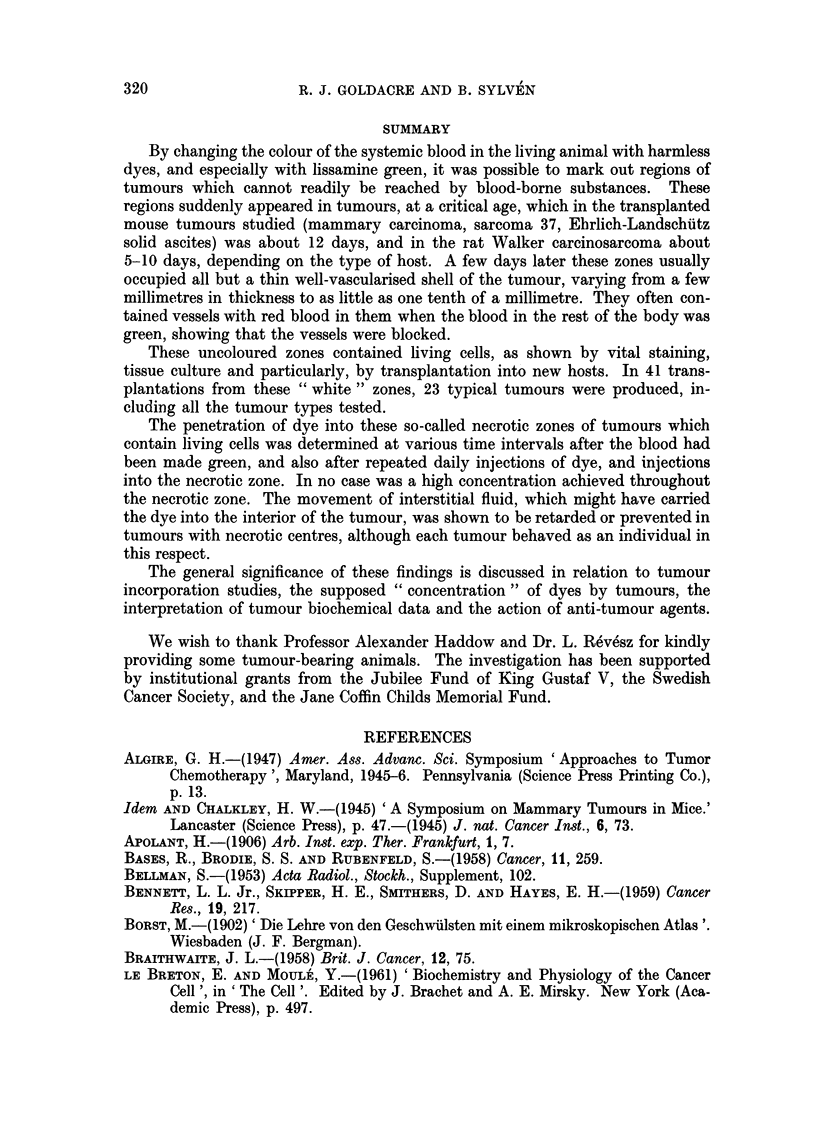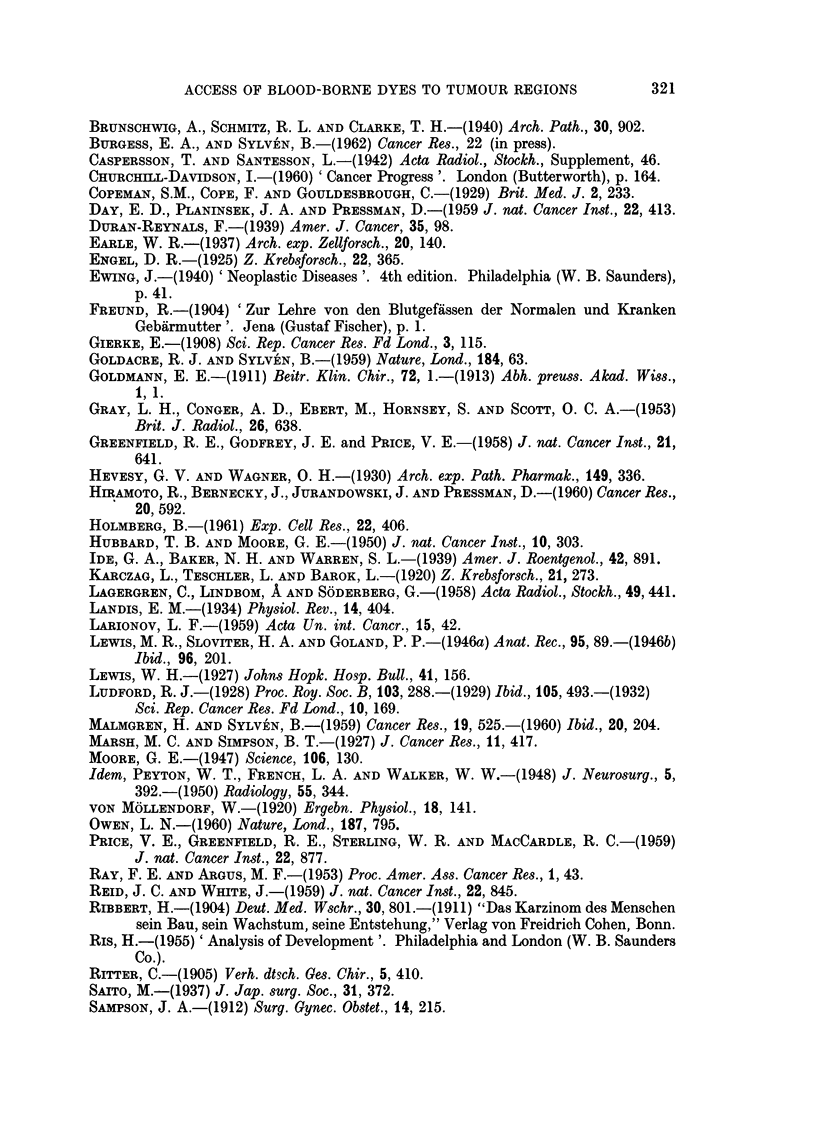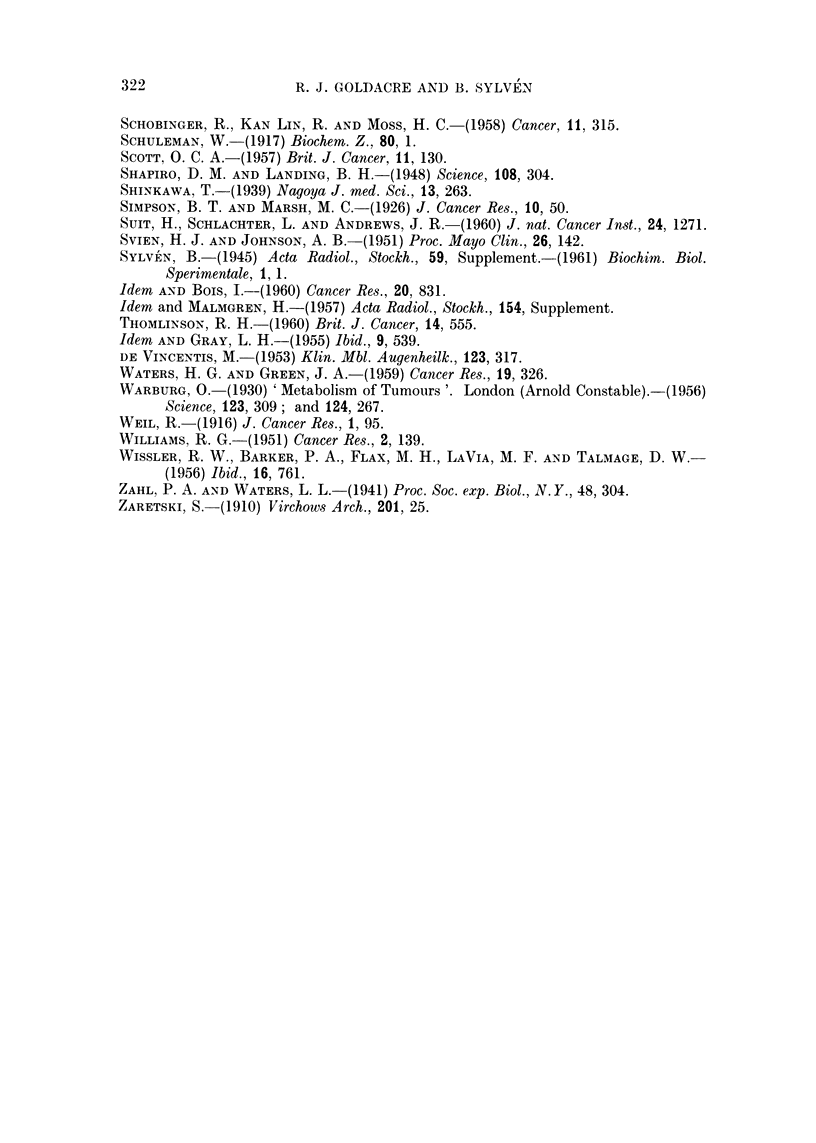# On the Access of Blood-Borne Dyes to Various Tumour Regions

**DOI:** 10.1038/bjc.1962.36

**Published:** 1962-06

**Authors:** R. J. Goldacre, B. Sylvén

## Abstract

**Images:**


### ON THE ACCESS OF BLOOD-BORNE DYES TO VARIOUS TUMOUR REGIONS

R. J. GOLDACRE AND B. SYLVÉN

*From the Cancer Research Division of Radiumhemmet, Karolinska Institutet, Stockholm 60 and the Chester Beatty Research Institute, London, S.W.3*

Received for publication April 14, 1962

THE degree of blood supply in vital and non-vital tumour regions has long been of interest in tumour biology in its various aspects. In this paper evidence is presented that many tumours contain substantial regions which cannot readily be reached by blood-borne substances, and that these regions contain living cells capable of starting tumours when transplanted into new hosts. These regions were easily mapped out by changing the colour of the systemic blood with a harmless dye which, in addition, coloured the interstitial fluid, but did not enter the living cells (Goldacre, 1955, unpublished data ; Goldacre and Sylvén, 1959; Holmberg, 1961). Preliminary data (Goldacre and Sylvén, 1959) indicated that in some tumours in rats and mice the only region presenting an open connection with the systemic circulation was a thin peripheral zone varying from a few millimetres to a tenth of a millimetre in thickness or less. Somewhat similar observations were made by Owen (1960) in spontaneous tumours of cats and dogs, using the same method. The blood, often visible in the regions unreached by the dye, was blocked off from the general circulation. In the present communication more detailed evidence on this point will be presented and the biological implications discussed.

The observations are mainly limited to certain commonly-used unicentric transplanted tumours. The conclusions do not necessarily apply to all other malignant tumours, since the regional vascular patterns are highly variable according to their individual mode of growth, malignancy, degree of invasiveness, destructiveness, and so on.

### HISTORICAL CONSIDERATIONS

The vascular morphology of spontaneous and transplanted tumours has been frequently commented upon and different concepts seem to have been accepted from time to time. For example, Borst (1902) Ribbert (1904) and Apolant (1906) concluded that most spontaneous malignant tumours were poorly and irregularly supplied with blood vessels particularly in central regions, and also that the existing vessels showed changes, such as defective coatings, dilatation, obliteration, thrombosis, etc. Among the transplantable tumours the so-called haemorrhagic mice mammary tumours attracted special attention (Apolant, 1906 ; Gierke, 1908) due to vascular rupture and interstitial bleeding. It was felt that the nutritional conditions of tumours became considerably poorer with the continued growth of the tumour (cf. Ewing, 1940). On the other hand these pathologists often noted that the vascular density at and around the tumour periphery was increased and that to some extent new formation of vessels had occurred. Later on and possibly as result of experience with transplanted tumours, the concept is met that solid tumours seemed to have a satisfactory blood supply or even a supply superior to the normal tissue (Algire and Chalkley, 1945), and a " preferred nutritional status ".

One of the first careful attempts to ascertain whether intratumoral vessels were patent to the flowing blood was made by Goldman (1911) using intra-arterial injections of indian ink in living animals. He described how small transplants were richly supplied with open vessels throughout, while larger tumours with central necrosis presented a vascular network only at the periphery. A more sparse and irregular net of capillary-like vessels was found in the interior of such tumours, many being obliterated by " eine fortschreitende spezifische Wanddegeneration ", as well as by active invasion of carcinoma cells (Freund, 1904) and conjoined thrombosis. Similar results have been reported by later investigators all using coloured or radio-opaque insoluble materials for the direct or roentgenological visualization of the arterial and/or venous supply of tumours of different kinds (Sampson, 1912 ; Lewis, 1927 ; Schobinger, Kan Lin and Moss, 1958 ; Braithwaite, 1958 ; Waters and Green, 1959). Extensive angiographic studies have been made by Japanese workers, notably Saito (1937) and Shinkawa (1939). The latter reported a marked increase in arterial blood supply during the early stages of growth of transplanted fowl sarcomas, and later a decrease due to obliteration and necrosis. His paper also contains an extensive list of angiographic references from 1919 to 1932. It might be noted that the resolving power of the usual angiographic procedure is rather low ; more recent micro-angiographic approaches (Bellman, 1953 ; Lagergren, Lindbom and Söderberg, 1958) could be applied to the tumour field.

More detailed data, particularly on the early vascular patterns of transplanted tumours, stem from *in vivo* observations with the transparent chamber technique. Ide, Baker and Warren (1939) in their study of the growth of explanted Brown-Pearce rabbit epitheliomas commented upon the high degree of vascularity in the peripheral parts of the tumour : the apparent avascularity in the necrotic centres had to do, they thought, with the intratumoural pressure resulting in vascular compression. The admirable works of Algire and Chalkley (1945) and Algire (1947) on different stages of tumour vascularization are well known. They observed that tumours in their early stages of growth induced a vascular proliferation, resulting in a vascular level of up to double that of the surrounding normal tissues ; they also noted at later stages that large sinusoids and blood-filled divertculi ended blindly towards the tumour centres as if they had been obliterated. These authors further corroborated the old observation that the new-formed tumour vessels had a single-layered endothelial wall lacking a more resistant external coating. The V2 rabbit carcinoma was studied by similar techniques by Williams (1951), who noticed that the established tumours completely obliterated their central vascular channels by external pressure, which led to central necrosis. The only surviving part of the explants was a narrow rim at the periphery where the host vessels had not yet been affected by the tumour.

Another heterogeneous group of observations refers to studies on the tissue distribution of coloured and fluorescent substances readily penetrating the capillary boundary thereby reaching the interstitial fluid compartment. These observations will thus not specifically refer to the vascular supply only, unless the distribution was recorded immediately following the systemic administration of the dye in question. These investigations were mostly performed with the aim of finding a " selective up-take " or " concentration " by tumour tissue to form a basis for further chemotherapeutic trials. As early as 1916 Weil observed that certain dyes, such as Congo red, remained in necrotic tumour regions after the dye had been excreted from the rest of the body, probably due to " a retarded rate of absorption from these poorly vascularized areas ". Shortly afterwards Karczag, Teschler and Barok (1920) and Engel (1925) applied light green (lissamine green), trypan blue and other dyes to tumour-bearing animals by subcutaneous injection. They noted some colouration of the peripheral tumour zone, but since the dye concentration in the blood was low after such subcutaneous injection the findings related more to the long-time uptake by stromal cells, as reported by Ludford (1928, 1929), than to the immediate vascular distribution. Ludford (1932) showed that trypan blue was taken up by stromal cells and not by tumour cells. In a variety of spontaneous and transplantable tumours Duran-Reynals (1939) described how Evans blue, 24 to 48 hours following intravenous injection of a small amount, appeared to be concentrated in the peripheral growing tumour regions, not reaching the necrotic zones. A similar dye distribution was reported by Brunschwig, Schmitz and Clarke (1940) in some human tumours, but benign tumours did not retain the dye 24 hours after the injection of a relatively small amount of Evan's blue, although only a small amount of dye was used compared with the amounts of dye used in the present study.

A more careful study of the distribution of Evans blue and other dyes at higher blood concentrations in mouse sarcoma 180 and a mammary carcinoma was performed by Zahl and Waters (1941). They observed an initial diffuse " staining " of the peripheral stroma and a more intense staining, confined to a thin layer, of " semi-necrotic cells " on the border of the viable and necrotic zones of the tumour. A selective uptake of Nile blue and other dyes by tumours was claimed by Lewis, Sloviter and Goland (1946). A localization of fluorescein in various tumours was found, and in brain tumours indicates an opening-up of the blood-brain barrier (Moore, 1947 ; Moore *et al.*, 1948, 1950 ; Hubbard and Moore, 1950 ; Svien and Johnson, 1951). A large number of additional references may be cited on the alleged concentration of dyes and other materials by tumours (Weil, 1916 ; Karczag *et al.*, 1920 ; Copeman, Cope and Gouldesbrough, 1929 ; Engel, 1925 ; Simpson and Marsh, 1926 ; Marsh and Simpson, 1927 ; Hevesy and Wagner, 1930 ; Brunschwig, Schmitz and Clarke, 1940 ; Ray and Argus, 1953 ; de Vincentis, 1953 ; Wissler *et al.*, 1956 ; Bases, Brodie and Rubenfeld, 1958 ; Reid and White, 1959). It will, however, be shown in the discussion that the dye is never concentrated in the tumours. Instead, it is more slowly washed out from the tumours than from the rest of the body, and never reaches a higher effective concentration (or chemical potential) in the tumour than that initially in the normal tissues. This conclusion is further substantiated by the critical study of the passage of fluorescein in Sarcoma 180 (Shapiro and Landing, 1948).

Another much debated question, the origin of tumour necrosis, is still not answered. It appears that most pathologists favour a two-fold mechanism : a destruction of vascular walls by tumour cells and obliteration by pressure from the surrounding tumour tissue (cf. above). Many microscopic data give evidence of the first mentioned cause, while some experimental observations (Williams, 1951) tend to support the latter possibility. Other factors could perhaps also play a role. Ribbert (1904, 1911) mentioned that the decrease in capillary pressure at the tumour periphery might account for the apparent deficiency in central nutrition leading to necrosis. What really happens to the vessels and circulation once the vessels have become surrounded by the tumour has not been as widely discussed as the new-formation and dilatation of vessels at the tumour periphery.

No data have been found in the literature directly referring to the *passage* of dyes and other molecules through the interstitial or extracellular compartment of tumours, but the *composition* of the interstitial fluid of some transplanted tumours has recently been reported (Sylvén and Bois, 1960; Burgess and Sylvén, 1962).

### MATERIALS AND METHODS

*Tumours and methods of transplantation.*—Most observations refer to commonly used unicentric transplants of the following mouse and rat tumours, all of which were rapidly growing types: Sarcoma 37 propagated in albino stock mice, ABC mammary carcinomas in ABC and CBA inbred mice and their hybrids, solid transplants of the hyperdiploid Ehrlich-Landschütz (ELD) carcinoma in both inbred and non-inbred stock mice, and the Walker carcino-sarcoma grown in Wistar rats (Chester Beatty strain) or domestic Swedish stock rats. In addition, some spontaneous mammary carcinomas in ABC mice and a few methylcholanthrene-induced mouse sarcomas and benzpyrene-induced rat sarcomas were used. In all, 150 tumours were examined. All transplants from solid tumours were made subcutaneously or intramuscularly with the usual trochar technique, while in a few cases ELD ascites tumour cell suspensions were injected into the legs of mice in order to produce multicentric tumours. Several transplants studied under a transparent window according to the Sandison-Clark technique will not be reported in detail since only the peripheral tumour vessels were easily observed.

*The dye.*—Various vital dyes were tried and the results with all were similar, but most of the work was done with the triphenylmethane dye lissamine green V (Gurr: Soc. Dyers and Colourists Colour Index No. 735). The closely related lissamine green B (C.I. No. 737) and light green SF (C.I. No. 670) were found to be equally suitable. This dye had special advantages over the others, having more contrast with the blood and being less toxic and less reactive than, for instance, trypan blue and Evans blue. Eosin and neutral red were unsuitable due to insufficient contrast with the blood, and methylene blue because it was decolourized in most tissues; however, it was possible to obtain results with these dyes by making them visible with further treatment (ammonia with neutral red, ultraviolet light with eosin and reoxidation in the air with methylene blue). Some results were also obtained with fluorescein (in conjunction with an ultraviolet lamp) which has been used extensively for diagnostic purposes on human patients by Moore and his colleagues (Moore, 1947; Moore *et al.*, 1948; Hubbard and Moore, 1950; Svien and Johnson, 1951). In addition, indian ink was used, but it usually killed the animals in amounts which made any appreciable difference to the colour of the blood; in lower amounts it could be seen in the microscope as black particles, usually agglutinated, in those blood vessels in which it was present. In general, 0·5–1 ml. of a 2 per cent lissamine green (LG) solution was rapidly injected into the tail vein, and this gave within a few seconds a peak of blood concentration of about 0·5 per cent in a 20 g. mouse. This dye is itself not decolourized by normal or tumour tissue during its passage through the animals nor during incubation with cell suspensions. In some experiments, Evans blue, eosin and trypan blue were also used alone or in combination with previous injections of LG.

The rate of interstitial movement of the green dye was measured with an optical micrometer, the site of injection being marked with indian ink which was added to the solution. Interstitial deposition of small dye drops around and inside the tumours in living mice under nembutal was made using a 27-gauge needle controlled by the dissecting microscope.

*Dissection and methods of observation.*—The green coloured regions of tumours were observed at various times after dye injection and immediately after killing the animal by exsanguination under ether. The path of circulating blood at the tumour periphery was observed sometimes within a few seconds after the injection before the dye had spread into the interstitial fluid, in living mice either injected with dye and immediately killed, or injected following dissection deeply into the tumour or else in tumours growing under a transparent window. In general, however, we chose to give the dye about one hour's time to spread before the animals were sacrificed and the tumours bisected. The extent of dye penetration was easily observed with a dissecting microscope. In some cases the dye was injected first after a subcutaneous tumour had been partially uncovered in order to allow miscoscopic inspection of the peritumoural vessels. Photographic recording of the topographical dye distribution was made using colour film.

### RESULTS

### (a) *Dye distribution and elimination in normal animals*

LG injection into the tail vein of normal mice resulted in a deep green colouration of the circulating blood volume and, within a few seconds, a bright green colour of the whole animal, except those organs protected by blood barriers (*vide infra*). It could be seen under the dissecting microscope that visible amounts of the green dye had moved out through the finest vessels into the extracellular fluid less than 30 seconds after the injection (cf. Landis, 1934). This movement of dye is of importance for the understanding of the tumour experiments.

The elimination via the urine and bile started shortly after the injection; the whole animal was in general cleared and regained its normal colour within 12–18 hours. During the elimination time the kidneys and liver appeared an intense dark green colour.

Living cells are not permeable to this dye-stuff as shown by *in vitro* tests (Goldacre, 1955, unpublished data; Goldacre and Sylvén, 1959; Holmberg, 1961). Hence most of the dye is free and mobile in the interstitial fluid and remains a good indicator of its movements. On the other hand, dead cells were instantaneously stained diffusely throughout their cytoplasm and nucleus by LG. No interstitial compartment in normal tissue retained the dye beyond 24 hours, even after intraperitoneal injections of dye solution. It was, however, noticed that a small amount of dye was retained by macrophages in peritoneal lymph plaques, possibly after capture by phagocytosis (cf. Schuleman, 1917; von Möllendorff, 1920).

The most pronounced transport barrier to this dye was observed in the brain (cf. Goldmann, 1913) which remained normal in colour as did the cerebrospinal fluid and spinal medulla. Other parts of mice and rats not reached by the green dye were the interior of the eye, the foetal structures including the foetal side of the placenta (cf. trypan blue; Zaretzki, 1910), and to a large extent the testes, ovary and adrenal glands, and occasionally some blood depots in the spleen and lungs in animals killed a few minutes after injection. The bone marrow distribution of dye was not studied. The blood-brain barrier also excluded from the brain the following dyes when present in the blood : trypan blue (cf. Goldmann, 1913), Evans blue, eosin and fluorescein, but not methylene blue and neutral red. In contrast, as mentioned in the next section, neither of these dyes entered in 1 hour the necrotic zones of the tumours.

(b) *The dye distribution in unicentric solid tumour transplants up to one hour after the injection*

The distribution of green dye in the tumours was found to be essentially the same whether the tumours were dissected 1–5 minutes after an intravenous injection or one hour after either intravenous or intraperitoneal injection. For each type of tumour the critical factor causing differences in the distribution of dye was mainly the age and to some extent the size of the tumours. Young tumours up to 12 days old (which in mice were usually less than 10 mm. across) were instantaneously coloured throughout as green as normal tissue (Table I). Once the tumours had reached a critical age, necrotic foci developed (Fig. 1–7). Some tumours with multifocal necrosis showed a green and white mottled appearance in central regions (Fig. 2) surrounded by a green periphery. In large tumours it was most striking to observe, under the microscope, that still patent vessels containing

TABLE I.—*Age of Tumours Showing Various Appearances in the Lissamine Green Test*

| | Age, in days, of tumours appearing in lissamine green test as : | | |
|---|---|---|---|
| Tumour | All green | Thick green periphery, small white centre | Thin green periphery, large white centre |
| Walker carcinosarcoma (rats) | | 5†; 10; 10<br>11; 13; 14. | 7†; 9†; 9*†; 11†; 14*; 14;<br>17R; 17*; 18*. |
| Solid ascites (ELD) | 6; 7;<br>8; 10;<br>12; 12. | 11; 12; 14. | 13*; 14*; 18; 21; 23; 23;<br>23; 32; 32R; 32; 32; 36;<br>36. |
| Sarcoma 37 | 6. | 11; 12; 14. | 12; 14; 14; 15; 16; 18*;<br>18; 19; 20; 20*; 21; 21;<br>22 (mottled); 24; 25; 29;<br>32; 32R; 33R; 33R; 33R;<br>42R; 42R; |
| Mammary carcinoma | 6. | 10; 13; 14; 14;<br>14; 15; 15. | 12; 12; 12; 13; 13; 13;<br>13; 14; 14; 14; 14; 14;<br>14; 14; 15; 15; 15; 15;<br>15; 15; 15; 16; 16; 17;<br>18; 18; 18; 18; 18; 18;<br>18; 18; 18; 18; 19; 19;<br>19; 19; 19; 19; 19; 19;<br>19; 19*; 20; 20*; 20*; 20;<br>20; 20; 20; 20; 20; 20;<br>20; 20; 20; 22; 22; 22;<br>22; 24; 28; 34. |

* In these tumours the green region was less than ½ mm. thick over most of the periphery of the tumour (measured with optical micrometer).

† Rapidly-growing tumour in Chester Beatty strain of Wistar rats ; the remaining Walker tumours were slower-growing transplants into a Stockhold strain of rats.

R indicates a regressing tumour.

bright red blood (Fig. 4 and 5) with intact blood cells were present in the white tumour region, while the blood in the rest of the body was dark green. In many large tumours one was surprised at the extreme thinness of the zone coloured by the dye as compared with the complete penetration of other tissues. The way in which the thickness of the green peripheral zone varied can be seen in Fig. 1–7. Similar findings were made with all the other dyes tested.

Multicentric tumours, including spontaneous mammary carcinomas and some methylcholanthrene induced sarcomas, showed a mottled appearance of green-stained rings surrounding different white centres of necrosis, or otherwise quite irregular patterns of green and white central areas.

(c) *The dye distribution six hours and later after the injection*

As previously mentioned the dye-stuff was macroscopically cleared from the body in 12 to 18 hours' time after the injection (Table II). After about 6 hours

TABLE II.—*Effect of Time on the Distribution of Lissamine Green ; 1 ml. 2 per cent Solution Injected Intraperitoneally ; cf. Fig. 8 and 9*

| Time (hours) | Depths of green colour in: Skin (in white mice) | Blood (from heart, macroscopic appearance) | Tumour: Centre | Tumour: Periphery |
|---|---|---|---|---|
| 1 . | Dark green | Deep Green | White | Green |
| 3 . | Deep green | Black-brown | White | Green |
| 6 . | Deep green | Dark red | White | Green |
| 9 . | Green | Red | Green ring around centre | White |
| 12 . | Pale green | Red | Green ring | White |
| 15 . | Very pale green | Red | Green ring | White |
| 18 . | White | Red | Green ring | White |
| 24 . | White | Red | Green, or green ring | White |
| 168 . | White | Red | Green | White |

EXPLANATION OF PLATES

FIG. 1.—Fifteen-day-old mammary carcinoma, bisected, in mouse 1 hour after LG injection, showing green normal tissue and green peripheral ring of tumour surrounding necrotic centre of normal yellow-red appearance. Note green skin, especially ears, paws and eyes.

FIG. 2.—Bisected mottled tumour ; mammary carcinoma, 15 days old, 1 hour after I.P. injection of L.G.

FIG. 3.—Mammary carcinoma, 15 days old, 1 hour after I.P. injection of LG, showing a fairly broad green peripheral zone, several millimetres thick.

FIG. 4*a* AND 4*b*.—Twenty-day-old mammary carcinoma 2 minutes after I.V. injection of LG. a and b : high power at different parts of same tumour periphery. The vascularised zone in this case is only about 100 $\mu$ thick. Tumour of vital appearance with red blood vessels occurs on the inside of this zone. The more central white zone, in places radiating out to the periphery, is completely necrotic.

FIG. 5.—Mammary carcinoma, 19 days old, 45 minutes after I.P. injection of LG, showing in most places broad vascularised zone with sharp borderline towards white centre. The white zone contains still patent vessels with red blood and intact red cells, while the rest of the blood in the body is dark green.

FIG. 6.—Multicentric tumour : bisected 28-day-old mammary carcinoma showing several foci of necrosis. I.P. injection of LG 1 hour before.

FIG. 7.—Solid multicentric Ehrlich-Landschütz (hyperdiploid) ascites tumour filling up almost the whole thigh of a mouse, showing the vast extent of the non-vascularised region. Thirty-two-day-old tumour, 5 minutes after I.V. injection of LG.

FIG. 8.—Twenty-day-old mammary carcinoma, bisected 24 hours after I.V. injection of LG, showing the green ring surrounding the necrotic centre. Outside the green ring note the ring of growing vital tumour tissue, which has been cleared of dye as has the rest of the body.

1

2

3

4a

4b

5

6

7

8

Goldacre and Sylvén.

the blood regained its normal colour to the eye. It would of course be expected that the dye would be washed out of the tumours in the same way provided that the tumours had equally effective transport facilities as normal organs, and that no strong dye adsorption occurred. This was so with young tumours lacking a necrotic region. They regained their natural colour within 24 hours after the injection.

In contrast to this, older tumours with a necrotic centre retained a green shell of dye in the region bordering the necrotic zone. The picture presented by the

FIG. 9.—Development of green ring. Diagram showing dye distribution in a tumour with a necrotic centre at various time intervals after injection of the dye. Not to scale. The three different appearances of the 24-hour stage illustrate the individuality of different tumours.

bisected tumours between 6 to 24 hours after the injection was that of a green ring with white growing tumour tissue outside it and necrotic tissue within the ring (Fig. 8 and 9).

The white tumour zone outside the green ring now constituted the growing well-vascularized zone. At 12 hours after the injection the thickness of this green ring in a 25 × 18 mm. sized mammary carcinoma was about 2–3 mm. (Fig. 9). Both the necrotic zone and the vital zone outside it advanced, in the mouse tumours used, at the rate of 1–1·5 mm. per day ; hence, a second injection of the same green dye a day later only resulted in a second green ring just outside the first one without increasing the depth of colour. Additional evidence of the retention of dye was obtained by injections of dyes of different colours on successive days. The first injection of LG was followed a day later by eosin, and still a day later by

Evans blue, after which the mice were killed within an hour's time. In this way concentric rings of three different colours were produced. The last blue ring largely represented the vascularized zone at the time of the last injection.

This experiment with 3 dyes makes clear why successive daily injections of the same substance did not increase the concentration in the necrotic zone of the tumour—they merely produced successive concentric shells of equally dyed tumour tissue. Later necrotic regions appeared as white shells outside the green ones. The dye retained in the centre was mostly bound to dead cells, which could be taken out and inspected under the microscope. In some tumours the green colour in the necrotic zone was not confined to the periphery after 24 hours, but extended throughout the necrotic zone (though much paler than the vital zone had been at the time of the injection) ; this was not a regular occurrence and each tumour appeared to behave as an individual in this respect. When it occurs, it seems to indicate some movement of interstitial fluid. Further information on such movement is given later.

The extent of penetration of dead tissue by diffusion was studied in separate experiments with boiled pieces of liver and kidney. These were implanted under the skin of mice, which were then injected intravenously with LG. The pieces were later cut across and found to have a green rind about 200 $\mu$ thick after 3 hours and 400–600 $\mu$ thick after 24 hours in the living mouse.

(d) *Various supplementary observations*

(1) *Injection into centre of tumour.*—In order to determine whether dye injected into the centre of tumours with necrotic centres could be carried to those parts of the tumour not reached by dye given by the intravenous or intraperitoneal route, concentrated dye solutions were injected into the centre of some large tumours. It was possible to inject up to a maximum of 0·1 ml. LG solution into the centre of medium-sized and large mouse tumours 2–3 weeks of age without dye flowing back through the puncture track. When such tumours were opened one day later the liquid dye solution was found still free in the centre of the tumour and along the needle track. Only those parts of the central detritus in contact with green solution were stained. The dye had not penetrated into other parts of the tumour and none appeared in the systemic circulation. The vital vascularized peripheral zone was always free of dye. Even in large Walker tumours rich in central fluid, the dye remained locally around the site of injection. When a cyst was injected, liquid in adjacent cysts did not become green and the cysts appeared not to be in communication with one another.

Attempts were made to increase the dye penetration still further by an injection first into the tumour centre followed 1–24 hours later by an intravenous or intraperitoneal injection. Tumours bisected 1 hour afterwards presented large white areas free of dye particularly in the important zone just inside the peripheral growing layer. The vitality of this intermediate unstained region is discussed below.

(2) *Interstitial deposition of dye.*—Dye deposited close to the distal surface of tumours always in a short time skirted along the tumour periphery and then followed the regular lymphatics away from the tumour site. In a few mammary carcinomas measurements of speed were made and found to be of the order of about 300 $\mu$ per minute. Dye from the outside never entered the subcortical layers of these tumours. Similarly, dye deposited in the tumour centres never leaked out through the tumour periphery.

These results again show the shielded-off character of medium- and large-sized tumours ; only the peripheral vascularized parts apparently have adequate interstitial transport of fluid.

(e) *Cytology and vitality of regions not reached by systemic blood-borne dyes*

One might assume that tumour regions not provided with flowing blood would contain only dead or injured cells unable to propagate in other locations. The external part of the non-vascularized solid tumour region just inside the periphery contains the older large-sized tumour cell generations of the so-called extreme " B " type near incipient necrosis (cf. Caspersson and Santesson, 1942) with a characteristic cytology and very low amounts of cytoplasmic ultra-violet absorbing materials. Distinctive enzymatic differences have been reported between the older cell generation and the younger growing ones at the tumour periphery called "*A*" cells (Sylvén and Malmgren, 1957 ; Malmgren and Sylvén, 1959, 1960). It is not yet known to what extent these old cells are vital or irreversibly damaged (cf. review by Sylvén, 1961). It seemed evident from LG tests for vitality that lack of blood supply did not necessarily lead to immediate cell death since living tumour cells were found in the central necrotic fluid of large Walker tumours. Living leukocytes were similarly found in the centre of experimentally induced non-vascularized abscesses. The following evidence further indicates that the non-vascularized tumour regions contain substantial numbers of vital cells.

(1) The lissamine green test for cell vitality showed that cells dissected from the central necrotic regions were mostly dead and those from the green periphery were all alive, while those from the intermediate zone were a mixed population of living and dead ones.

(2) Several mice carrying tranplanted tumours 16 days old with a diameter of about 15 mm. were injected with LG intravenously (1 ml. of 1 per cent solution) in order to mark out the peripheral vascularized region, and killed in two minutes. From representative blocks through the whole tumours and the surrounding tissue, fresh frozen serial sections were cut in the cryostat and inspected under a cover slip with a microscope. Thick 70 micron sections gave sufficiently intense green colour to allow identification of the border line between the green and white region (cf. above). Alternate 12 micron sections were cut, fixed and stained with Azure A for histology. Several significant findings were made.

The uniform green colour of the tumour periphery faded away at its inner border within a few dozen microns ; next followed a region about 130 $\mu$ thick containing only living cells with only a trace of dye invisible to the eye. Inside this there was a thin green line of dead cells (Fig. 10), strongly adsorbing that trace of dye which had diffused across the 150 $\mu$ layer of living cells. This stained layer has also been described by Zahl and Waters (1941) in mouse sarcoma 180 and mammary carcinoma 15091a immediately after the intravenous injection of Evans blue, but they did not, however, observe the thin uncoloured layer outside it. The outermost uniformly coloured zone thus represents the well-vascularized region having immediate access to flowing blood. The supply of dye to the next zone of living tumour tissue, about 150 $\mu$ thick, is largely effected by diffusion from this zone outside it. The local concentration of dye is further influenced by its adsorption at the necrotic border.

Now, the question arises whether living cells still remain inside this green border line. Microscopic inspection of thin stained serial sections mentioned above did reveal groups of tumour cells of vital appearance at a distance of up to 2 mm. inside the sharp green border line.

These groups of living tumour cells occurred as islands at various places in the necrotic region, and were not in connection with the blood stream, since the green dye had not advanced so far. Their isolation from the peripheral shell of vital tumour tissue was further shown by following their extent in thick serial sections for about 2 mm. of the tumour slices. Their cytoplasmic basophilia,

FIG. 10.—Appearance of thick (70 μ) radial section across green-white border of tumour slice, 2 minutes after I.V. injection of LG. Not to scale.

greater than that of typical *B* cells, and their nuclear characteristics suggested that they might be vital (cf. Caspersson and Santesson, 1942; Sylvén and Malmgren, 1957).

(3) *Tissue culture.*—Pieces of tumour tissue from the region of tumours not supplied with flowing blood were dissected out of 15- and 20-day old mammary carcinomas in mice. The mice had 1 hour previously been injected with LG, so that the green-white border provided a guide for the dissection.

Pieces just inside the green border were then explanted in hanging drop cultures, using horse plasma and chick embryo extract. A sparse outgrowth occurred from the white pieces in 5 to 8 days, as compared with a profuse outgrowth from the green pieces in only 2 days. This suggests the presence of living cells also in the necrotic part of the tumours, cells which *in vitro* are able to move out of the dead material.

(4) *Transplantation experiments.*—Similarly trimmed small pieces from the non-vascularised white region of large mammary carcinomas and sarcoma 37 in mice were transplanted into new hosts of the same strains. Similarly samples of uncoloured fluid pipetted from Walker tumours, in rats previously injected with

LG as a guide, were implanted in 0·5 ml. amounts subcutaneously in new hosts. In order to be sure that no contamination from peripheral tumour zones could occur some Walker tumours were opened up by means of a diathermy knife. From 41 transplantations made, 23 typical tumours were obtained, and verified as tumours in a representative sample by their histology. This clearly shows that the necrotic zones do contain living cells able to propagate the tumours (Table III).

TABLE III.—*Results of Transplantation Experiments*

| | | | | | | | | |
|---|---|---|---|---|---|---|---|---|
| WALKER central fluid, | positive | takes | in | 14 | out | of | 25 | cases |
| MAMMARY CARCINOMA | ,, | ,, | ,, | 7 | ,, | | 12 | ,, |
| SARCOMA 37 | ,, | ,, | ,, | 2 | ,, | | 4 | ,, |

The evidence thus indicates that the dye did not reach all the living tumour cells in one hour's time. Only a slow dye diffusion will occur into these non-vascularized regions where nests of still vital cells have been demonstrated.

(f) *Notes on the vascular histology*

In 12–70 $\mu$ thick fresh cryostat sections a rich vascular network of all sizes down to near capillary dimensions was noted only in the peripheral region. Most vessels followed across the green-white border ended abruptly due to a kind of collapse. Many vessels could not be followed further, others became transformed into a thin cord-like hyaline structure. In the necrotic regions all kinds of deranged vessels were noted similar to those observed by Ritter (1905) and other pathologists, such as sinusoidal channels, vessels lacking a visible membrane, and others bordered by tumour cell vegetations, in which the coverings had perished. Most of these vessels contained still undamaged red blood cells, and represented the occluded channels no longer in connection with the systemic circulation as previously described under Results, Sections (b) and (c), and in Fig. 4*a*, 4*b* and 5. In the white necrotic zones of some tumours, protein deposits were seen presenting a positive Weigert's fibrin stain (cf. Hiramoto *et al.*, 1960). This is further evidence of the marked vascular destructiveness of these tumours.

### DISCUSSION

Our present results corroborate the general view that young and small transplanted tumours are well vascularised ; however, further light is thrown on the blood supply of larger tumours, and of the vitality of various regions in them, and on the transport of substances in and out of tumours.

After our unicentric tumours reached a size of about 10 mm. in diameter, corresponding to an age, in the mouse tumours, of about 12 days, a central necrotic region appeared, which did not become green up to one hour after the intravenous dye injection, and carried no flowing blood. Nevertheless, this region often contained still patent vessels containing intact red cells. Since the dye did not reach these vessels in our experiments, the conclusion is drawn that they were occluded at some place. The detailed mechanism of occlusion and obliteration of vessels, which illustrates the destructiveness of the tumour, remains to be elucidated ; some of the biochemical and physical factors involved have been discussed by Sylvén (1945), Sylvén and Malmgren (1957), Sylvén and Bois (1960) and Burgess, Bois and Sylvén (1962). The destructive activity of the tumour cells on the vessel walls, widely reported in the literature, would account for the blockage of vessels at the green-white border and their subsequent transformation into a hyaline core and ultimate complete removal. This will also explain the trapping and destruction, in some places, of erythrocytes and haemoglobin (Greenfield, Godfrey and Price, 1958 ; Price *et al.*, 1959) as well as the occurrence of fibrin deposits in the stromal compartment of tumours (Day, Planinsek and Pressman, 1959 ; Hiramoto *et al.*, 1960). This destructive activity is also directed against other components in the stroma, such as muscle and collagen fibres (Sylvén, 1945 ; Burgess, Bois and Sylvén, 1962 ; Sylvén and Malmgren, 1957).

In order to explain the green ring which becomes apparent on the periphery of the necrosis after the dye has been mainly excreted from the body (i.e. after one day, though some indication is present in 6 hours, Fig. 9), we have to consider that the blood-borne dye rapidly penetrates the vessel walls and reaches the interstitial tumour fluid. In the living zone the dye is not adsorbed on the cells but instead washed out again after about 12–24 hours (Table II). In contrast, however, when the dye reaches the first layer of dead cells it is bound there as shown in Fig. 10. The width of this zone will increase with time owing to the advance of the edge of the necrotic zone (about 1·5 mm. per day) during the time the dye is present in the blood in sufficient concentration to stain dead tissue on the edge of this zone ; and its width will also increase to some extent by diffusion.

The amount of dye in the green ring is partly influenced by its adsorption on denatured proteins, and this might be quite different with other substances in the blood which are not adsorbed or are normally incorporated (sugars, amino acids, nucleotides, etc.). But most of the dye captured in the ring would be due to the lack of transport out of the necrotic zone, for the vascular zone recedes beyond the reach of effective diffusion into it from most parts of the green ring, owing to the widening of the necrotic zone with time.

The dye thus captured by the necrotic zone of the tumour, and remaining there after it has been excreted from the rest of the body, appears to have given rise to the widespread but erroneous statement in the literature that tumours " concentrate " a wide variety of dyes (Marsh and Simpson, 1927 ; de Vincentis, 1953) and other substances (Hevesy and Wagner, 1930 ; Wissler *et al.*, 1956). Where trypan blue was truly captured Ludford (1929) showed that the dye was actually in RES cells at the tumour periphery and not in the tumour cells themselves.

The observed vascular blockage further implies that the force driving the interstitial fluid will drop to zero beyond the blockage. The observations indicate that interstitial flow of dye in the tumour periphery is as great as that in normal tissues surrounding the tumours. On the other hand, in the non-vascularized tumour zone interstitial transport seems negligible, these parts having little or no lymph flow as indicated by the failure of dye solutions injected into the tumour centres to disappear in a short time as it does in normal tissues (see Section d under Results).

The condition of stasis seems to furnish part of the explanation for the high content in the central tumour fluid, as compared with normal interstitial fluid, of proteins and enzymes (Sylvén and Bois, 1960) and various metabolites, including lactate (Burgess, Bois and Sylvén, 1962). Further, the reduced transport of interstitial fluid will influence the rate of incorporation of blood-borne substances (cf. Bennett *et al.*, 1959) and the interpretation of autoradiographic data (Reid and White, 1959) on solid tumours similar to those used in the present paper.

In those tumour regions where there is a lack of blood supply, there will be various interesting consequences. There will naturally be some necrosis, as described by Borst (1902), Ribbert (1904), Ritter (1905), Goldman (1911), Lewis (1927) and others ; our present results give further evidence on the extent to which it occurs, and the rather unexpected result that all tumour cells do not die, at least for some considerable time, in the complete absence of a blood supply. The abrupt fall in oxygen tension from the peripheral zone to the non-vascularised zone, over a range of a few hundred microns (Fig. 10, layer C) means that only in the periphery can the cells have an oxygen supply adequate for aerobic conditions, while in the interior any still vital cells remaining would have a nearly anaerobic medium influenced by the rate of oxygen diffusion as well as by the metabolic activity of the local cell populations (cf. the Erlang-Krogh equation and calculations on tumour material by Thomlinson and Gray, 1955). However, absence of oxygen alone is not sufficient to cause death of tumour cells, for it is well known from the work of Warburg (1930, 1956) and others that tumour cells can survive in the complete absence of oxygen by switching over to anaerobic glycolysis, which is a characteristic of all types of cancer cells (le Breton and Moulé, 1961). Such death as occurs must therefore result from the lack of other blood-borne metabolites and/or the accumulation of metabolic or autolysis products which would normally be removed by the blood or lymph. Some nutrients for a proportion of the tumour cells could probably be provided by the death and autolysis of other tumour cells around them (Earle, 1937 ; Ris, 1955). The cytology and growth potential of the remaining living cells would probably be altered by this changed medium, which would contain both nutrient and noxious substances.

The drop in oxygen tension would also influence the cellular radio-sensitivity as discussed by Gray *et al.* (1953), Scott (1957), Churchill-Davidson (1960), Thomlinson (1960), and Suit, Schlachter and Andrews (1960). It is noteworthy that Thomlinson (1960) has also concluded, from X-ray studies of tumours in high pressures of oxygen, that tumour cells survived in necrotic centres ; the extra oxygen only increased the radiosensitivity of cells outside the necrotic regions. A similar interpretation could be placed on the work of Suit, Schlachter and Andrews (1960), who reported that tumours 10–15 mm. across did not respond better to X-rays in two atmospheres pressure of oxygen than in air, whereas tumours 7–10 mm. across did.

The results reported above have a bearing on attempts to control the growth of solid tumours by means of blood-borne chemicals and anti-tumour sera. The access of a drug to all the cells of a solid tumour would depend on the presence or absence of a necrotic centre in the tumour, which in turn depends upon the age and size of the tumour in the way we have already indicated. This is consistent with the statement by Larionov (1959) based on clinical material that " the degree of anti-tumour effect is inversely proportional to the mass of the tumour tissue ".

Our experiments lead us to conclude that, in the tumours we have studied, the necrotic centre is an uneven dispersion of living tumour cells surviving almost anaerobically in a medium of autolysed tumour tissue, which has no blood supply and exchanges material only very slowly with the external living tissue.

SUMMARY

By changing the colour of the systemic blood in the living animal with harmless dyes, and especially with lissamine green, it was possible to mark out regions of tumours which cannot readily be reached by blood-borne substances. These regions suddenly appeared in tumours, at a critical age, which in the transplanted mouse tumours studied (mammary carcinoma, sarcoma 37, Ehrlich-Landschütz solid ascites) was about 12 days, and in the rat Walker carcinosarcoma about 5–10 days, depending on the type of host. A few days later these zones usually occupied all but a thin well-vascularised shell of the tumour, varying from a few millimetres in thickness to as little as one tenth of a millimetre. They often contained vessels with red blood in them when the blood in the rest of the body was green, showing that the vessels were blocked.

These uncoloured zones contained living cells, as shown by vital staining, tissue culture and particularly, by transplantation into new hosts. In 41 transplantations from these " white " zones, 23 typical tumours were produced, including all the tumour types tested.

The penetration of dye into these so-called necrotic zones of tumours which contain living cells was determined at various time intervals after the blood had been made green, and also after repeated daily injections of dye, and injections into the necrotic zone. In no case was a high concentration achieved throughout the necrotic zone. The movement of interstitial fluid, which might have carried the dye into the interior of the tumour, was shown to be retarded or prevented in tumours with necrotic centres, although each tumour behaved as an individual in this respect.

The general significance of these findings is discussed in relation to tumour incorporation studies, the supposed " concentration " of dyes by tumours, the interpretation of tumour biochemical data and the action of anti-tumour agents.

We wish to thank Professor Alexander Haddow and Dr. L. Révész for kindly providing some tumour-bearing animals. The investigation has been supported by institutional grants from the Jubilee Fund of King Gustaf V, the Swedish Cancer Society, and the Jane Coffin Childs Memorial Fund.

### REFERENCES

ALGIRE, G. H.—(1947) *Amer. Ass. Advanc. Sci.* Symposium ' Approaches to Tumor Chemotherapy ', Maryland, 1945–6. Pennsylvania (Science Press Printing Co.), p. 13.

*Idem* AND CHALKLEY, H. W.—(1945) ' A Symposium on Mammary Tumours in Mice.' Lancaster (Science Press), p. 47.—(1945) *J. nat. Cancer Inst.*, **6**, 73.

APOLANT, H.—(1906) *Arb. Inst. exp. Ther. Frankfurt*, **1**, 7.

BASES, R., BRODIE, S. S. AND RUBENFELD, S.—(1958) *Cancer*, **11**, 259.

BELLMAN, S.—(1953) *Acta Radiol., Stockh.*, Supplement, 102.

BENNETT, L. L. Jr., SKIPPER, H. E., SMITHERS, D. AND HAYES, E. H.—(1959) *Cancer Res.*, **19**, 217.

BORST, M.—(1902) ' Die Lehre von den Geschwülsten mit einem mikroskopischen Atlas '. Wiesbaden (J. F. Bergman).

BRAITHWAITE, J. L.—(1958) *Brit. J. Cancer*, **12**, 75.

LE BRETON, E. AND MOULÉ, Y.—(1961) ' Biochemistry and Physiology of the Cancer Cell ', in ' The Cell '. Edited by J. Brachet and A. E. Mirsky. New York (Academic Press), p. 497.

BRUNSCHWIG, A., SCHMITZ, R. L. AND CLARKE, T. H.—(1940) *Arch. Path.*, **30**, 902.
BURGESS, E. A., AND SYLVÉN, B.—(1962) *Cancer Res.*, 22 (in press).
CASPERSSON, T. AND SANTESSON, L.—(1942) *Acta Radiol., Stockh.*, Supplement, 46.
CHURCHILL-DAVIDSON, I.—(1960) ' Cancer Progress '. London (Butterworth), p. 164.
COPEMAN, S.M., COPE, F. AND GOULDESBROUGH, C.—(1929) *Brit. Med. J.* **2**, 233.
DAY, E. D., PLANINSEK, J. A. AND PRESSMAN, D.—(1959 *J. nat. Cancer Inst.*, **22**, 413.
DURAN-REYNALS, F.—(1939) *Amer. J. Cancer*, **35**, 98.
EARLE, W. R.—(1937) *Arch. exp. Zellforsch.*, **20**, 140.
ENGEL, D. R.—(1925) *Z. Krebsforsch.*, **22**, 365.
EWING, J.—(1940) ' Neoplastic Diseases '. 4th edition. Philadelphia (W. B. Saunders), p. 41.
FREUND, R.—(1904) ' Zur Lehre von den Blutgefässen der Normalen und Kranken Gebärmutter '. Jena (Gustaf Fischer), p. 1.
GIERKE, E.—(1908) *Sci. Rep. Cancer Res. Fd Lond.*, **3**, 115.
GOLDACRE, R. J. AND SYLVÉN, B.—(1959) *Nature, Lond.*, **184**, 63.
GOLDMANN, E. E.—(1911) *Beitr. Klin. Chir.*, **72**, 1.—(1913) *Abh. preuss. Akad. Wiss.*, **1**, 1.
GRAY, L. H., CONGER, A. D., EBERT, M., HORNSEY, S. AND SCOTT, O. C. A.—(1953) *Brit. J. Radiol.*, **26**, 638.
GREENFIELD, R. E., GODFREY, J. E. and PRICE, V. E.—(1958) *J. nat. Cancer Inst.*, **21**, 641.
HEVESY, G. V. AND WAGNER, O. H.—(1930) *Arch. exp. Path. Pharmak.*, **149**, 336.
HIRAMOTO, R., BERNECKY, J., JURANDOWSKI, J. AND PRESSMAN, D.—(1960) *Cancer Res.*, **20**, 592.
HOLMBERG, B.—(1961) *Exp. Cell Res.*, **22**, 406.
HUBBARD, T. B. AND MOORE, G. E.—(1950) *J. nat. Cancer Inst.*, **10**, 303.
IDE, G. A., BAKER, N. H. AND WARREN, S. L.—(1939) *Amer. J. Roentgenol.*, **42**, 891.
KARCZAG, L., TESCHLER, L. AND BAROK, L.—(1920) *Z. Krebsforsch.*, **21**, 273.
LAGERGREN, C., LINDBOM, Å AND SÖDERBERG, G.—(1958) *Acta Radiol., Stockh.*, **49**, 441.
LANDIS, E. M.—(1934) *Physiol. Rev.*, **14**, 404.
LARIONOV, L. F.—(1959) *Acta Un. int. Cancr.*, **15**, 42.
LEWIS, M. R., SLOVITER, H. A. AND GOLAND, P. P.—(1946*a*) *Anat. Rec.*, **95**, 89.—(1946*b*) *Ibid.*, **96**, 201.
LEWIS, W. H.—(1927) *Johns Hopk. Hosp. Bull.*, **41**, 156.
LUDFORD, R. J.—(1928) *Proc. Roy. Soc. B*, **103**, 288.—(1929) *Ibid.*, **105**, 493.—(1932) *Sci. Rep. Cancer Res. Fd Lond.*, **10**, 169.
MALMGREN, H. AND SYLVÉN, B.—(1959) *Cancer Res.*, **19**, 525.—(1960) *Ibid.*, **20**, 204.
MARSH, M. C. AND SIMPSON, B. T.—(1927) *J. Cancer Res.*, **11**, 417.
MOORE, G. E.—(1947) *Science*, **106**, 130.
*Idem*, PEYTON, W. T., FRENCH, L. A. AND WALKER, W. W.—(1948) *J. Neurosurg.*, **5**, 392.—(1950) *Radiology*, **55**, 344.
VON MÖLLENDORF, W.—(1920) *Ergebn. Physiol.*, **18**, 141.
OWEN, L. N.—(1960) *Nature, Lond.*, **187**, 795.
PRICE, V. E., GREENFIELD, R. E., STERLING, W. R. AND MACCARDLE, R. C.—(1959) *J. nat. Cancer Inst.*, **22**, 877.
RAY, F. E. AND ARGUS, M. F.—(1953) *Proc. Amer. Ass. Cancer Res.*, **1**, 43.
REID, J. C. AND WHITE, J.—(1959) *J. nat. Cancer Inst.*, **22**, 845.
RIBBERT, H.—(1904) *Deut. Med. Wschr.*, **30**, 801.—(1911) "Das Karzinom des Menschen sein Bau, sein Wachstum, seine Entstehung," Verlag von Freidrich Cohen, Bonn.
RIS, H.—(1955) ' Analysis of Development '. Philadelphia and London (W. B. Saunders Co.).
RITTER, C.—(1905) *Verh. dtsch. Ges. Chir.*, **5**, 410.
SAITO, M.—(1937) *J. Jap. surg. Soc.*, **31**, 372.
SAMPSON, J. A.—(1912) *Surg. Gynec. Obstet.*, **14**, 215.

SCHOBINGER, R., KAN LIN, R. AND MOSS, H. C.—(1958) *Cancer*, **11**, 315.
SCHULEMAN, W.—(1917) *Biochem. Z.*, **80**, 1.
SCOTT, O. C. A.—(1957) *Brit. J. Cancer*, **11**, 130.
SHAPIRO, D. M. AND LANDING, B. H.—(1948) *Science*, **108**, 304.
SHINKAWA, T.—(1939) *Nagoya J. med. Sci.*, **13**, 263.
SIMPSON, B. T. AND MARSH, M. C.—(1926) *J. Cancer Res.*, **10**, 50.
SUIT, H., SCHLACHTER, L. AND ANDREWS, J. R.—(1960) *J. nat. Cancer Inst.*, **24**, 1271.
SVIEN, H. J. AND JOHNSON, A. B.—(1951) *Proc. Mayo Clin.*, **26**, 142.
SYLVÉN, B.—(1945) *Acta Radiol.*, *Stockh.*, **59**, Supplement.—(1961) *Biochim. Biol. Sperimentale*, **1**, 1.
*Idem* AND BOIS, I.—(1960) *Cancer Res.*, **20**, 831.
*Idem* and MALMGREN, H.—(1957) *Acta Radiol.*, *Stockh.*, **154**, Supplement.
THOMLINSON, R. H.—(1960) *Brit. J. Cancer*, **14**, 555.
*Idem* AND GRAY, L. H.—(1955) *Ibid.*, **9**, 539.
DE VINCENTIS, M.—(1953) *Klin. Mbl. Augenheilk.*, **123**, 317.
WATERS, H. G. AND GREEN, J. A.—(1959) *Cancer Res.*, **19**, 326.
WARBURG, O.—(1930) ' Metabolism of Tumours '. London (Arnold Constable).—(1956) *Science*, **123**, 309 ; and **124**, 267.
WEIL, R.—(1916) *J. Cancer Res.*, **1**, 95.
WILLIAMS, R. G.—(1951) *Cancer Res.*, **2**, 139.
WISSLER, R. W., BARKER, P. A., FLAX, M. H., LAVIA, M. F. AND TALMAGE, D. W.—(1956) *Ibid.*, **16**, 761.
ZAHL, P. A. AND WATERS, L. L.—(1941) *Proc. Soc. exp. Biol.*, *N.Y.*, 48, 304.
ZARETSKI, S.—(1910) *Virchows Arch.*, **201**, 25.